# Supplementary material for: Graph-theoretical formulation of the generalized epitope-based vaccine design problem
Source: PLoS Comput Biol. 2020 Oct 23;16(10):e1008237. doi: 10.1371/journal.pcbi.1008237 (PMC7652351; doi:10.1371/journal.pcbi.1008237)
Supplement: S1 Appendix — Section A contains a brief description of the ϵ-constrain method [34] to obtain Pareto-efficient solutions in a bi-objective optimization problem, while section B contains the procedure to quantify conserved, low variability pathogen regions in terms of entropy on aligned sequences. (PDF) [file pcbi.1008237.s001.pdf]

A Obtaining Pareto-efficient solutions in a multi-objective optimization problem

We wish to explore the Pareto frontier of this bi-objective optimization problem (for simplicity, we assume there is only a single subset P):

$$\begin{aligned} \text{Maximize} \quad & z = \left\{ I(P) - \sum_{e_{ij} \in H(P)} w(e_{ij}) \right\} \\ \text{Subject to} \quad & |P| \leq k \end{aligned} \tag{1}$$

One possible algorithm to do so is the augmented ϵ-constraint method [?], an iterative solving approach in which one of the two objectives is moved into the constraints and bounded to take values smaller than e_i:

$$\begin{aligned} \text{Maximize} \quad & I(P) - \epsilon s \\ \text{Subject to} \quad & \sum_{e_{ij} \in H(P)} w(e_{ij}) - s = e_i \\ & |P| \leq k, s \in \mathbb{R}^+ \end{aligned} \tag{2}$$

with ϵ small. The surplus variable s is used in the objective function to discard weakly efficient solutions, when multiple optimal solutions with the same immunogenicity exist. The bounds $e_1, \ldots, e_N$ uniformly span the range between

$$\min_{|P| \leq k} \sum_{e_{ij} \in H(P)} w(e_{ij}) \tag{3}$$

and

$$\min_{|P| \leq k} \sum_{e_{ij} \in H(P)} w(e_{ij}) \text{ s.t. } I(P) = \max_{|P| \leq k} I(P) \tag{4}$$

i.e., the minimum edge cost, and the minimum edge cost with the maximum unconstrained immunogenicity. This procedure requires to solve $N + 3$ instances of the problem and results in N points on the Pareto frontier balancing the immunogenicity and the cumulative cleavage likelihood.

B Measuring residue conservation in terms of entropy

We quantified the variation in each position of the pathogen by computing the entropy on aligned sequences, only in the position where the consensus was not a gap. Valdar showed that the entropy at a given position is an acceptable metric to quantify residue conservation, and, indirectly, its importance for the structure and function of the protein [?]. The entropy of the i-th residue is computed as:

$$H_i = -\sum_a \frac{n_{ai}}{N} \log \left(\frac{n_{ai}}{N} \right) \tag{5}$$

with n_{ai} counting how many aligned sequences contain the amino acid a in position i, and N the number of sequences. Since we are interested in the conservation of nine-mers, we smooth the entropy using a triangular filter of width 9:

$$\tilde{H}_i = \frac{1}{25} \sum_{j=-4}^{4} (5 - |j|) H_{i+j} \tag{6}$$

References

[1] Mavrotas G. Effective implementation of the ϵ-constraint method in Multi-Objective Mathematical Programming problems. Applied Mathematics and Computation. 2009;213(2):455–465. doi:10.1016/j.amc.2009.03.037.

[2] Valdar WSJ. Scoring Residue Conservation. Proteins: Structure, Function, and Genetics. 2002;48(2):227–241. doi:10.1002/prot.10146.
