# Supplementary material for: Graph-theoretical formulation of the generalized epitope-based vaccine design problem
Source: PLoS Comput Biol. 2020 Oct 23;16(10):e1008237. doi: 10.1371/journal.pcbi.1008237 (PMC7652351; doi:10.1371/journal.pcbi.1008237)
Supplement: S2 Appendix — This appendix contains the two alternative formulations of the ILP where average epitope conservation and pathogen coverage are maximized together with immunogenicity. (PDF) [file pcbi.1008237.s002.pdf]

Maximize		
(OBJ)	$\sum_{t\in T}\sum_{v\in V}\sum_{a\in A} y_{vt}p_a i_{va} + \sum_{s\in S}\theta_s$	
Subject to		
(C1)	$\sum_{v\in V} x_{wvt} = \sum_{v\in V} x_{vwt} = y_{wt}$	$\forall w \in V, t \in T$
(C2)	$\sum_{t\in T} y_{vt} \leq 1$	$\forall v \in V$
(C3)	$\sum_{t\in T}\sum_{v\in V} x_{svt} = \sum_{t\in T}\sum_{v\in V} x_{vst} = \|t\|$	
(C4)	$\sum_{(v,w)\in E} x_{vwt} w(e_{vw}) \leq h$	$\forall t \in T$
(C5)	$\sum_{v\in V} y_{vt} \leq k$	$\forall t \in T$
(C6a)	$u_{vt} - u_{wt} + 1 \leq (\|v\| - 1)(1 - x_{vwt})$	$\forall e_{vw} \in E, t \in T$
(C6b)	$1 \leq u_{vt} \leq \|v\| - 1$	$\forall v \in V, t \in T$
(C7)	$\sum_{t\in T}\sum_{v\in V} y_{vt}\tau_{vs} \geq \theta_s$	$\forall o \in S$
(C8)	$x_{vwt}, y_{vt}, \theta_s \in \{0, 1\}$	$\forall v, w, t, s$

Table A: Alternative ILP formulation where pathogen coverage is maximized together with immunogenicity.

Maximize		
(OBJ)	$\sum_{t \in T} \sum_{v \in V} \sum_{a \in A} y_{vt} p_a i_{va} + \sum_{t \in T} \sum_{v \in V} \sum_{s \in S} y_{vt} \tau_{vs}$	
Subject to		
(C1)	$\sum_{v \in V} x_{wvt} = \sum_{v \in V} x_{vwt} = y_{wt}$	$\forall w \in V, t \in T$
(C2)	$\sum_{t \in T} y_{vt} \leq 1$	$\forall v \in V$
(C3)	$\sum_{t \in T} \sum_{v \in V} x_{svt} = \sum_{t \in T} \sum_{v \in V} x_{vst} = \|T\|$	
(C4)	$\sum_{(v,w) \in E} x_{vwt} w(e_{vw}) \leq h$	$\forall t \in T$
(C5)	$\sum_{v \in V} y_{vt} \leq k$	$\forall t \in T$
(C6a)	$u_{vt} - u_{wt} + 1 \leq (\|v\| - 1)(1 - x_{vwt})$	$\forall e_{vw} \in E, t \in T$
(C6b)	$1 \leq u_{vt} \leq \|v\| - 1$	$\forall v \in V, t \in T$
(C7)	$x_{vwt}, y_{vt} \in \{0, 1\}$	$\forall v, w, t$

Table B: Alternative ILP formulation where epitope conservation is maximized together with immunogenicity.
