# Supplementary material for: Graph-theoretical formulation of the generalized epitope-based vaccine design problem
Source: PLoS Comput Biol. 2020 Oct 23;16(10):e1008237. doi: 10.1371/journal.pcbi.1008237 (PMC7652351; doi:10.1371/journal.pcbi.1008237)
Supplement: S1 Table — This appendix contains a table listing the 27 MHC alleles used in this study and their percent frequency in the world population. (PDF) [file pcbi.1008237.s006.pdf]

Allele	Freq.	Allele	Freq.	Allele	Freq.
A*01:01	4.50	A*02:01	10.69	A*02:05	0.88
A*03:01	3.69	A*11:01	7.52	A*24:02	12.91
A*31:01	2.43	A*68:01	1.77	B*07:02	3.61
B*08:01	2.95	B*15:01	2.06	B*27:02	0.15
B*27:05	1.11	B*35:01	3.24	B*37:01	0.44
B*38:01	0.66	B*39:01	1.77	B*40:01	5.31
B*40:06	0.52	B*44:03	2.21	B*51:01	3.24
B*51:02	0.22	B*52:01	0.88	B*58:01	2.65
C*04:01	8.26	C*06:02	5.09	C*07:02	9.66

Table A: HLA alleles and their percent frequency in the world population used in this study.
