# Supplementary material for: Graph-theoretical formulation of the generalized epitope-based vaccine design problem
Source: PLoS Comput Biol. 2020 Oct 23;16(10):e1008237. doi: 10.1371/journal.pcbi.1008237 (PMC7652351; doi:10.1371/journal.pcbi.1008237)
Supplement: Attachment — Submitted filename: review_PLOSCompBio_April2020.pdf [file pcbi.1008237.s007.pdf]

The manuscript describes an interesting generalization of epitope vaccine design and derives a unifying formalism for three frequently used epitope vaccine design methods. The general idea is very interesting, however - partly due to its generality - it is very hard to follow and understand. Parts of the manuscript seem poorly written (figures and legends are incomplete, typos, needed explanations are missing, etc.), and generally speaking, the manuscript is written to be only understood by experts in the respective field. Many details that may seem obvious to the authors but aren't to non-experts are left out and need to be better explained in a revised version.
I highly encourage the authors to seek colleagues who work in computational science and/or HIV vaccinology to proof-read an edited version before resubmission. This will help the authors to fill in the gaps needed to make a more general computational scientist and/or HIV researcher able to appreciate their work.

Major:

1. 89ff: The assumption that each epitope contributes independently to the vaccine?s overall immunogenicity seems unrealistic. Given its importance for the relevance of the proposed method, the authors should comment on how realistic it is rather than just referring to Toussaint et al.

2. I missed (in the Discussion) a discussion of the general (non-technical) limitations of this work. What may prevent actual experimental HIV vaccinologists from using this framework? What assumptions are not/not exactly true in reality? Answers to questions like this are missing...

3. Why do you need to include s in your algorithm? Can't you just get rid of it and conceptually replace h by $h + a + b$? My intuition is that s just unnecessarily complicates things.

4. Fig 3: Five bootstrap values, which values were used? This is not described anywhere. Also, in the text (l168(you cannot write "For each of the five bootstraps" without even mentioning Fig. 3 or where the bootstraps were used and what values were used. The way the whole subsection "Jointly approaching epitope selection and assembly captures trade-off between cleavage likelihood and immunogenicity" is written at the moment is extremely unsatisfying. Explicit numbers appear out of nowhere, it is unclear where they come from and then a conclusion appears that can also not be independently validated given what is provided. A similar example of explicit numbers appearing without explanation is line 192: 216 amino acids. Or line 283, "Some of the previous results were obtained by only considering 300 random proteins out 283 of a few thousand", which ones remains unclear.

5. Where do the 2000 epitopes come from? How were they selected? The methods only describe the set of HIV-1 sequences used as a running example throughout the manuscript. Also, why did you choose to use only subtype B and C sequences and "only" 2271? Shouldn't you use all available subtypes and a much larger number of sequences as a potential HIV vaccine should not be subtype-specific and so that pathogen coverage becomes more meaningful?

6. Details are missing at many places in this manuscript; please add. The methods especially can/should be expanded.

Minor:

1. Legend Fig.2 is incomplete. F.e., what are the differently colored sets of vertices? This should become clear from the legend, not much later in the text.

2. 70: typo: 2x "the"

3. 71: The use of the word vaccine here needs to be further explained. Either here or prior to this.

4. 74: If all simple tours of $P_1, \ldots, P_n$ start and end at s, these sets can by definition not be disjoint. Similarly, if $s \in P_i$, the intersection of P_i and P_j cannot be empty. They share s. Maybe let $P_i \subset V$ instead of $\tilde{V}$? That would then also agree with what you state in Eq. (1).

5. When describing Fig.2 you should clarify the meaning of n as it is of key importance to the methodology.

6. Table 1: It would be nice to show the optionality of (C7) and (C8) in the table.
7. l185: weird, inaccurate wording: meeting extreme requirements. Please describe what you mean.
8. Table 1: How does (C3) ensure connectedness of each tour as stated in line 141? Also, same table, (C6): what is the meaning of $u_{..}$? Should this maybe be y?
9. Fig. 5: missing y-labels. A reader should always have a chance to understand a figure by itself. The legend is simply a help. Also missing % on y-ticks for (a)?
10. Fig. 6c: show the full name of each method or explain in the legend.
11. Fig. 7: show full names
12. l312: typo: principleS
13. l320: typo: ... obtain A guaranteed...
14. l355: The sentence "Therefore, the solver can be ..." is incomplete. Words missing?
15. l409: Explain better why only 93% coverage can be achieved. I can guess but it is not clear from the text.
16. more minor typos throughout the manuscript...
