# Supplementary material for: Graph-theoretical formulation of the generalized epitope-based vaccine design problem
Source: PLoS Comput Biol. 2020 Oct 23;16(10):e1008237. doi: 10.1371/journal.pcbi.1008237 (PMC7652351; doi:10.1371/journal.pcbi.1008237)
Supplement: Attachment — Submitted filename: replies.pdf [file pcbi.1008237.s008.pdf]

Reply to Reviewers

Dear Reviewers, we are grateful for your comments on our manuscript. Please find below our replies to your concerns.

Reviewer 1

Dear Reviewer 1,

We appreciate your comments and apologize for our inaccurate writing. We extensively rewrote the manuscript to improve its readability, asked two colleagues of ours with different backgrounds to read the manuscript, and integrated their suggestions.

We would like to inform you that the Pareto frontier in Fig. 3 changed significantly because we found and fixed a bug in an external library we used to compute the cleavage scores. In response to Reviewer 2's comments, we also extended that experiment to compare string-of-beads designed by joining epitopes directly and by using optimized spacers (line 209), and used the same results to show that ordering is indeed optimized by our framework (line 227).

> The assumption that each epitope contributes independently to the vaccine's overall immunogenicity seems unrealistic. Given its importance for the relevance of the proposed method, the authors should comment on how realistic it is rather than just referring to Toussaint et al.

We agree with your assessment and added a paragraph discussing this issue (line 524), explaining how the presence of certain epitopes can inhibit or suppress the response towards other epitopes that would normally be recognized. The biology underlying this phenomenon and generally what factors impact vaccine immunogenicity are currently poorly understood and unpredictable, therefore, in absence of extensive experimental data and prior knowledge that can be integrated, we followed Occam's Razor and chose a linear function as immunogenicity. Also note that the framework is agnostic to the specific definition, and Toussaint's objective function could be easily replaced once our collective understanding of what constitutes a good vaccine improves. In fact, in response to Reviewer 2's comments, we added two new experiments (line 369) where we compared the results obtained with different immunogenicity predictors, and showed that there is no qualitative difference in the evaluation metrics we proposed.

> I missed (in the Discussion) a discussion of the general (non-technical) limitations of this work. What may prevent actual experimental HIV vaccinologists from using this framework? What assumptions are not/not exactly true in reality? Answers to questions like this are missing...

We added a paragraph explaining other important aspects that must be considered in the design of vaccines, but are not tackled by our framework (line 502). Different methods exist, each with its own advantages and disadvantages, to deliver the antigen designed with our framework to the relevant cells needed to initiate the immune response. Several mechanisms are used by pathogens to avoid or escape the immune system, and these should be taken into account as well. Finally, vaccines should generate a holistic response comprising both T- and B-cells, which can be done with our framework by using a suitable immunogenicity function. In short, many high-level decisions are left to the practitioner.

> Why do you need to include s in your algorithm? Can't you just get rid of it and conceptually replace h by h + a + b? My intuition is that s just unnecessarily complicates things.

That would be a valid formulation, but we think the current one is more appropriate. It corresponds to the team orienteering problem and highlights the equivalence of the two problems. It also enables users to directly employ heuristics or other solving methods designed for that problem in case our ILP proves too impractical to solve. See our references [23, 24].

> Fig 3: Five bootstrap values, which values were used? This is not described anywhere. Also, in the text (l168(you cannot write "For each of the five bootstraps" without even mentioning Fig. 3 or where the bootstraps were used and what values were used. The way the whole subsection "Jointly approaching epitope selection and assembly captures trade-off between cleavage likelihood and immunogenicity" is written at the moment is extremely unsatisfying. Explicit numbers appear out of nowhere, it is unclear where they come from and then a conclusion appears that can also not be independently validated given what is provided. A similar example of explicit numbers appearing without explanation is line 192: 216 amino acids. Or line 283, "Some of the previous results were obtained by only considering 300 random proteins out of a few thousand", which ones remains unclear.

We apologize for the inconvenience. We introduced the necessary details before these results, clarifying how the bootstraps were created. We also added all the sequences as supplement S3 and the sequences in each bootstrap in supplement S5. Furthermore, we tried to be more clear in how numbers in the results were computed.

> Where do the 2000 epitopes come from? How were they selected? The methods only describe the set of HIV-1 sequences used as a running example throughout the manuscript. Also, why did you choose to use only subtype B and C sequences and "only" 2271? Shouldn't you use all available subtypes and a much larger number of sequences as a potential HIV vaccine should not be subtype-specific and so that pathogen coverage becomes more meaningful?

We detailed how we chose the sequences (line 550). We indeed used all sequences matching our search filters, namely whole sequences more recent than 2003, which we chose to try to guarantee a certain minimum quality in the sequences themselves. Subtypes B and C cover the vast majority of cases in Europe, the Americas and Asia, while subtype A is mostly found in Africa. Pathogen coverage is, therefore, meaningful in those regions. We specified this in the relevant section (line 553). However, it might not be possible to design an effective global HIV vaccine due to its high mutation rate resulting in large epitope variability. In this case, a subtype-specific vaccine would be the way to go. We cannot answer these questions at present, which are open for further investigation by the practitioner.

> Details are missing at many places in this manuscript; please add. The methods especially can/should be expanded.

We added details on the sequence selection, as mentioned in the previous point, and computation of the edge weights for string-of-beads (line 574). We also added a paragraph listing the technologies we used to implement the framework (line 605).

In response to your minor comments, we corrected the formulation of Eq. 1 and the related explanations. In order to stay close to the original team orienteering formulation, we preferred to keep P_i as a subset of $\tilde{V}$. We also improved the explanation of several constraints in the ILP. We clarified the role of u in the Miller-Tucker-Zemlin constraints (line 166), how it eliminates subtours, and how it contributes to ensuring tour connectedness. We removed the abstract concept of options, and instantiated the coverage constraints separately for alleles and pathogens. We removed mentions that the constraints are optional, and explained in the table that the coverage/conservations parameters can be set to zero if those constraints are not required. We finally restyled the figures to make them easier to read, and amended wrong or missing elements therein.

Yours sincerely,
Emilio Dorigatti.

Reviewer 2

Dear Reviewer 2,

Thank you for your kind comments.

> The authors should discuss and guide the reader a bit more through the differences (or common features and applicability) of the presented model to other vaccine types such as the personalized therapeutic cancer vaccines or vaccines triggering the humoral immune response to produce antibodies.

We added a paragraph in the discussion (line 453) describing how the framework can be used in a personalized cancer setting, and how it can be used to generate cytotoxic T-cells or plasma cells as needed. All is needed is a reinterpretation of the components of the framework: for example, the epitope immunogenicity can be adapted to include B-cell epitopes too. In cancer vaccines, the somatic mutations would act as the pathogens from which epitopes are extracted, and with respect to which coverage would be computed. The patient's genotype would be the only HLA alleles that would need to be considered.

> The authors define the vaccine immunogenicity function vaguely as the ability to induce a broad immunization in a target population using sum of products from HLA allele frequencies times individual immunogenicity as predicted from NetMHCpan. For NetMHCpan was suggested to use '% rank' which is compared to a background set rather than 'IC50' as it is better comparable between different HLA types. The authors may provide more evidence that the effect of changing individual immunogenicity (MHC binding affinity) prediction using e.g. other prediction tools could lead to similar results and conclusions.

We added a new section (line 369) containing two experiments that showed no qualitative difference when different immunogenicity predictors were used (fig. 9 and 10). Specifically, we computed immunogenicities with the percentile rank from NetMHCpan and binding affinities from PickPocket and MHCflurry. We then repeated the pareto experiment and compared the optimized immunogenicity with the immunogenicity computed by the other methods, showing that they were very well correlated. We also repeated the experiment where we compare vaccines optimized for immunogenicity, conservation, and coverage, and showed that there is no qualitative difference among the different immunogenicity predictors.

> For the string-of-bead vaccines the question of spacers should be mentioned and discussed (see publication on BioRxiv by the same authors https://doi.org/10.1101/2020.04.25.060988)

We explained how the framework can be used to include spacers in string-of-bead EVs (line 591), and extended the pareto experiment with a comparison between string-of-beads when the epitopes are joined directly and when they are joined with optimized spacers (line 209). These spacers were designed before the application of our framework by using a method introduced by Schubert & Kohlbacher in 2016. We would also like to inform you that the Pareto frontier for the vaccines without spacers in Fig. 3 changed significantly because we found and fixed a bug in an external library we used to compute the cleavage scores. We finally added a discussion of the difference between the frameworks of the present paper and the bioRxiv preprint (line 600).

> Further should be studied and discussed whether the order of the epitopes have some effect on the results and conclusion.

We used the results of the Pareto experiment without spacers to empirically show that the framework also optimizes the ordering of the epitopes (line 227). We shuffled the epitopes on these vaccines and measured the consequent worsening of cleavage score, and explained why the vaccines on the frontier have the optimal ordering.

In response to your minor comments, we amended wrong or missing elements in the figures, and added a paragraph explaining the technologies we used to implement the framework, based on the Python ecosystem, and to solve the linear program, based on Pyomo and Gurobi (line 605).

Yours sincerely,
Emilio Dorigatti.
